# Children in Crisis: Addressing Nutrition Amid Conflict and Economic Hardship in Lebanon

**DOI:** 10.3389/ijph.2025.1608412

**Published:** 2025-03-07

**Authors:** Lamis Jomaa, Krystel Ouaijan, Lara Nasreddine

**Affiliations:** ^1^ Department of Nutrition, Gillings School of Global Public Health, University of North Carolina at Chapel Hill, Chapel Hill, NC, United States; ^2^ Department of Nutrition and Food Sciences, Faculty of Agricultural and Food Sciences, American University of Beirut, Beirut, Lebanon

**Keywords:** food security, nutrition, malnutrition, conflict, humanitarian crises, children, adolescents

Lebanon faces combined challenges from a persistent financial depression, political gridlock, and a devastating war that began in October 2023 and escalated to a nationwide crisis in September 2024. This escalation resulted in widespread destruction and the internal displacement of 899,725 individuals, of whom 7% were children under-five (CU5) and 27% were school-aged children and adolescents. Among the internally displaced, 21% have been forced to settle in collective shelters, having no other viable housing options [1]. These successive crises have significantly undermined access to basic needs and essential services, and disrupted livelihoods and security. In 2021, 58.7% of households reported food insecurity, and this rose to 68.2% in 2022 [2]. By 2024, 23% of the population, equivalent to 1.26 million individuals, were categorized in IPC-Phase 3 or higher, indicating a crisis that necessitates urgent humanitarian intervention [3].

The compounded crises in Lebanon have severely affected the most vulnerable population groups, including children and adolescents, disrupting their access to adequate nutrition, education, essential health services, and stable caregiving environments. Among infants, exclusive breastfeeding rates dropped from 32.4% in 2021 to 22.7% in 2024, [4, 5], and the 2023 national Lebanese Integrated Micronutrient, Anthropometry and Child Development (LIMA) survey showed that only 10% and 25.8% of CU5 are meeting the Minimum Acceptable Diet (MAD) and Minimum Dietary Diversity (MDD), respectively [4]. While wasting rates remain relatively low, with Global Acute Malnutrition reported at less than 2% in both 2021 and 2024, the prevalence of micronutrient deficiencies remains alarmingly high amongst CU5. Approximately 36% of children aged 6–59 months are iron-deficient, 16.9% are anemic, while 34.9% and 6.4% have zinc and vitamin A deficiencies, respectively [4]. These findings reflect the present and pressing risk of the hidden hunger, further underscored by a concerning rise in stunting rates from 10.4% in 2021 to 13.9% in 2024 [4, 5]. Although the focus in emergencies is typically on CU5 due to their rapid growth and developmental needs, adolescents and school-aged children—a population often overlooked in emergencies—are also heavily affected. In 2022, 54% of Lebanese adolescents reported experiencing food insecurity, while 37.6% of children aged 5–11 were classified as facing severe food insecurity, according to household-based surveys [2, 6]. The LIMA survey also showed that only 53.5% of adolescent girls met the MDD in 2023 and documented high rates of micronutrient deficiencies in this population group, including anemia (16.7%), vitamin D insufficiency (78.4%), and zinc deficiency (38.7%) [4].

In 2022, at a pivotal time when the country was witnessing an unprecedented economic crisis and a deterioration in food security, the Lebanese Ministry of Public Health launched its first National Nutrition Strategy and Action Plan (2021–2026) to help prioritize and ensure governance of nutrition interventions and coordinate efforts at the national level [7]. While this strategy prioritized maternal and young child nutrition, and the mitigation of micronutrient deficiencies and stunting, the recent war escalation has further set back the country and undermined efforts exerted by key national and global stakeholders to address these health and nutrition goals. At the same time, a call for strengthening strategic and technical coordination between UN agencies at local and regional levels was set forward by the Regional Nutrition Collaboration Framework to accelerate nutrition action in the Middle-East and North Africa (MENA) region [8]. The regional framework underscores the need for leveraging the systems of health, agriculture, social protection, education, and water, hygiene, and sanitation (WASH) to develop a holistic nutrition response aimed at improving nutrition among young children, school-aged children and adolescents. Yet, the adaptation and operationalization of this framework, and other global nutrition agendas [9, 10], in conflict-affected countries within the region are lacking, or limited, at best. The MENA region presents stark differences not only in political dimensions and conflict, but also in the burden and prevalent forms of malnutrition. High rates of obesity and associated diseases characterize affluent countries within the Gulf Cooperation Council, while countries undergoing constant turmoil such as Palestine, Lebanon, Syria, and Yemen grapple with the triple burden of malnutrition (undernutrition, micronutrient deficiency, and overnutrition). To protect the rights of all children and adolescents to healthy food and adequate nutrition, humanitarian efforts that include more agile response mechanisms and adaptable frameworks to address the complex emergencies witnessed in the region are needed today more than ever. With the looming climate crises and persistent geopolitical challenges expected to further contribute to regional and local conflicts, displacement, food insecurity, and adverse health outcomes, there is an imminent need to revisit local, regional, and global frameworks to promote nutrition action that is inclusive, equitable, achievable, and leaving no child behind.
